# Duodenal Malignant Somatostatinoma

**DOI:** 10.1155/1995/94629

**Published:** 1995

**Authors:** A. M. R. Ferrante, D. Frontera, G. B. Doglietto, G. de Santis, G. Viola, F. Crucitti

**Affiliations:** 1 Department of Surgery Catholic University of the Sacred Heart School of Medicine-Rome Italy; 2 Department of Surgery General Hospital-Offida (AP) Italy; 3 Istituto di Clinica Chirurgica Policlinico “A. Gemelli” L. go A. Gemelli, 8- Rome 00168 Italy

## Abstract

The authors report a case of hormonally silent duodenal somatostatinoma. The main clinical features, the natural history and the currently available therapies of these rare neoplasms are described on the basis of this case and of the scientific literature. Although the antiblastic therapies are still debated, the patient showed a surprising outcome following chemotherapy.


*HPB Surgery*, 1995, Vol. 8, pp. 263–266
Reprints available directly from the publisher
Photocopying permitted by license only

© 1995 OPA (Overseas Publishers Association)
Amsterdam B.V. Published in The Netherlands
by Harwood Academic Publishers GmbH
Printed in Malaysia

### CASE REPORT

### Duodenal Malignant Somatostatinoma

A. M. R. FERRANTE*, D. FRONTERA, G. B. DOGLIETTO, G. DE SANTIS[§], G. VIOLA and F. CRUCITTI

Department of Surgery, Catholic University of the Sacred Heart School of Medicine-Rome, Italy
[§] Department of Surgery, General Hospital-Offida (AP) Italy
* Istituto di Clinica Chirurgica, Policlinico "A. Gemelli" L. go A. Gemelli, 8-Rome 00168 Italy

*(Received October 23, 1993)*

The authors report a case of hormonally silent duodenal somatostatinoma. The main clinical features, the natural history and the currently available therapies of these rare neoplasms are described on the basis of this case and of the scientific literature. Although the antiblastic therapies are still debated, the patient showed a surprising outcome following chemotherapy.

KEY WORDS: Duodenal neoplasms somatostatinoma

### INTRODUCTION

Somatostatinoma is a neuroendocrine neoplasm derived from the D cells of the APUD system firstly described by Pearse[1]. This tumor is rare and the duodenal location has been described in less than 10 cases. Unlike pancreatic somatostatinoma, described in the late 70's[2,3], which generally produces a clinical syndrome (diabetes, steatorrhea, hypochloridria, dyspepsia), duodenal somatostatinoma may be completely asymptomatic.

We report a case of silent duodenal somatostatinoma with hepatic metastases that has been treated by chemotherapy obtaining a surprisingly steady response.

### CASE REPORT

The patient was a 43-year-old white woman with a pancreatic pseudocyst previously treated with a cystojejunostomy and a Y-en-Roux gastro-enterostomy and a past history of NANB viral hepatitis. She suffered from dyspepsia for about 1 year. Some months prior to admission the patient had undergone a gastroduodenoscopy because of the onset of post-prandial vomiting and epigastric pain radiating to the back. The endoscopist noticed a patent gastro-enterostomy and a marked dilatation of the duodenal bulb due to a stenosis in the second part of the duodenum.

On admission, the physical examination was completely negative. The general condition and the nutritional status were good. Routine blood analysis showed abnormal liver function tests (elevated alkaline phosphatase, SGPT and SGOT) and a mild anemia (Hb 12.7 g/dl); tumor markers (CEA, CA 19–9, CA 50, CA 125) were normal. A further endoscopy confirmed the duodenal stenosis and a biopsy was obtained; the histology suggested a neuroendocrine neoplasm and immunohistochemistry was positive for somatostatin, NSE and chromogranin. A plasma somatostatin level obtained was 285 pg/ml (normal range 70–150 pg/ml); urine 5–hidroxyindolacetic acid was slightly increased. Abdominal US showed two slightly hyperechogenic lesions (15 and 22 mm, respectively) with hypoechogenic edges in the right hepatic lobe and a solid, hypodense mass (3 cm in diameter) located medially and next to the duodenum.

A CT scan (Fig. 1) revealed duodenal dilatation with a mass protruding into the visceral lumen; hepatic masses showed an angiomatous appearance after the injection of contrast medium.

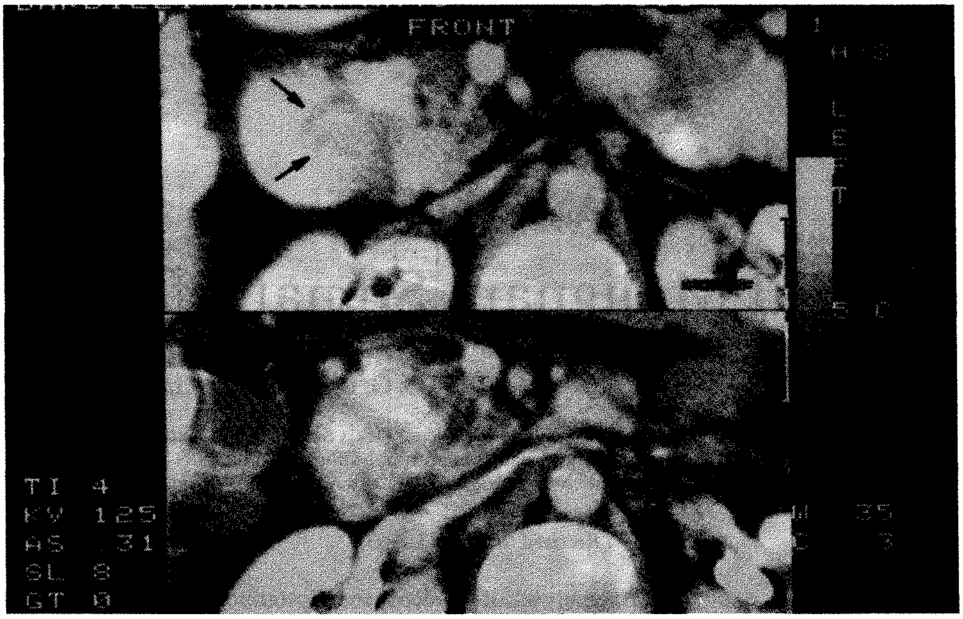

**Figure 1** Abdominal CT scan showing duodenal dilatation with a mass protruding into the visceral lumen.

A selective angiography of the celiac artery and its branches (Fig. 2) noticed a hypervascularized mass in the second part of the duodenum, containing micro arterovenous fistulae with stasis of the contrast medium; one of the hepatic lesions in the right lobe had a pathologic vascular appearance but not of an angiomatous type.

At laparotomy, multiple bilateral hepatic metastases were found. Frozen sections revealed metastases of apudoma and definitive immunohistochemistry (Fig. 3) confirmed the diagnosis of somatostatinoma. The postoperative period was uneventful. Before discharge, the patient underwent a $^{131}$I-MIBG scintiscan to evaluate the usefulness of radiometabolic therapy; the images obtained showed a dishomogeneous uptake which contraindicated nuclide treatment.

In the following months, ambulatory chemotherapy was given (calcium L-folinate 100 mg/m$^2$ and 5–FU 370 mg/m$^2$ i.v. for 5 days every 28 days, and $\alpha$–2b–Interferon 3,000,000 U subcutaneously on alternate days without interruption) for 8 cycles.

The comprehensive evaluation of the response to the therapy showed steady disease lasting 8 months. Infact, a further abdominal US revealed, 9 months after surgery, increased diameter of the hepatic metastases and chemotherapy was discontinued.

The patient is alive 24 months after diagnostic laparotomy; she is in good conditions and does not complain of clinical symptoms of somatostatinoma syndrome. She has undergone two further US scans that have not shown any further growth of the hepatic lesions. The levels of urine 5–hidroxyindolacetic acid and plasma somatostatin were in the normal ranges.

### DISCUSSION

Somatostatinoma has been more frequently observed in the pancreas, 45% of the described cases were located there. Duodenal location, first reported in 1979[4,5], is rare and accounts for 19% of the overall literature. This neoplasm may produce a clinicalsyndrome including diabetes, steatorrhea, hypochlorhidria and dyspepsia; cholelithiasis and anaemia have also been described[6]. However, it should be mentioned that in a recent review[7] no symptoms were specific enough to be considered as pathognomonic.

Duodenal somatostatinoma is hormonally "silent" in most cases[8]. High levels of other hormones (calcitonin, insulin, Pancreatic Polypeptide, VIP, ACTH, 5–HIAA) have been described in plasma or as cell immunoreactivity. The expanding neoplasm produces symptoms of alimentary tract obstruction (dyspepsia, duodenal stenosis, obstructive jaundice) so it may be

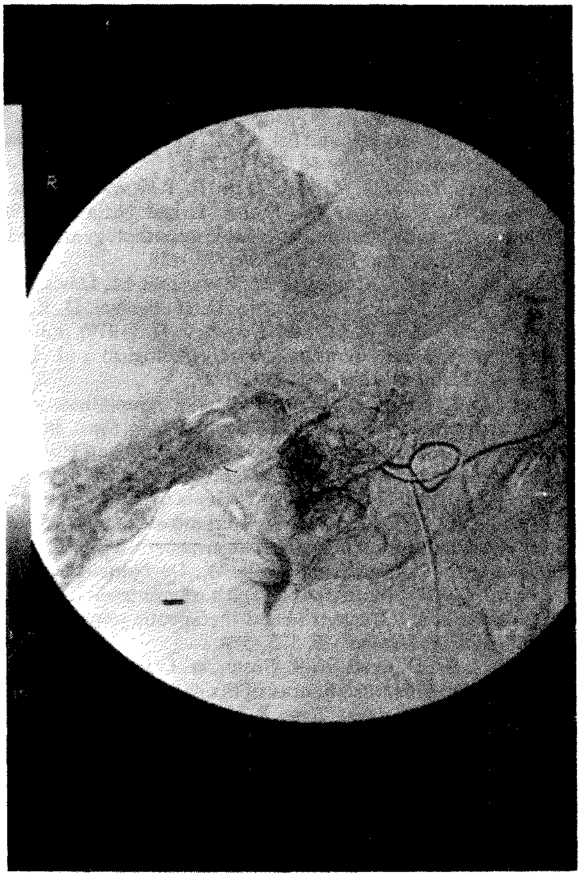

**Figure 2** Selective angiography of the celiac artery and its branches: a hypervascularized mass in the second part of the duodenum is evident. The neoplasm contains micro-arterovenous fistulae with stasis of the contrast medium.

undistinguishable from the more common adenocarcinoma. In our case, the previous surgery for pancreatic cyst may have been misleading in diagnostic evaluation. Moreover, diagnostic imaging techniques such as US, CT scan and endoscopy do not seem, in our experience, to give specific, results.

A rise in circulating levels of somatostatin following intravenous infusion of calcium and pentagastrin has been described[9] as a diagnostic test in patients affected by a "non functional" somatostatinoma, but is should be considered that this technique has been employed in already diagnosed cases, so its use as a "true" diagnostic tool seems questionable if there are not further elements suggestive of a somatostatinoma. Possibly because of slow growth and nonspecific symptoms, the diagnosis is often late, and metastases have been observed in 88% of cases of somatosatinoma[7]. Tumor spread frequently affects the liver (42%), as in our patient; other common sites involved are regional lymphnodes (39%) and the duodenum.

When technically feasible, surgical treatment seems to be the only effective therapy for this type of neoplasms; the most suitable procedure is a duodenopancreatectomy.

The role of adjuvant therapy is still debated, because of the paucity of reported experiences. However, a single more effective agent has not been defined to date, chemotherapy seems to be – as in our case – an useful tool for prolonged control of distant metastases[6,7,10,11].

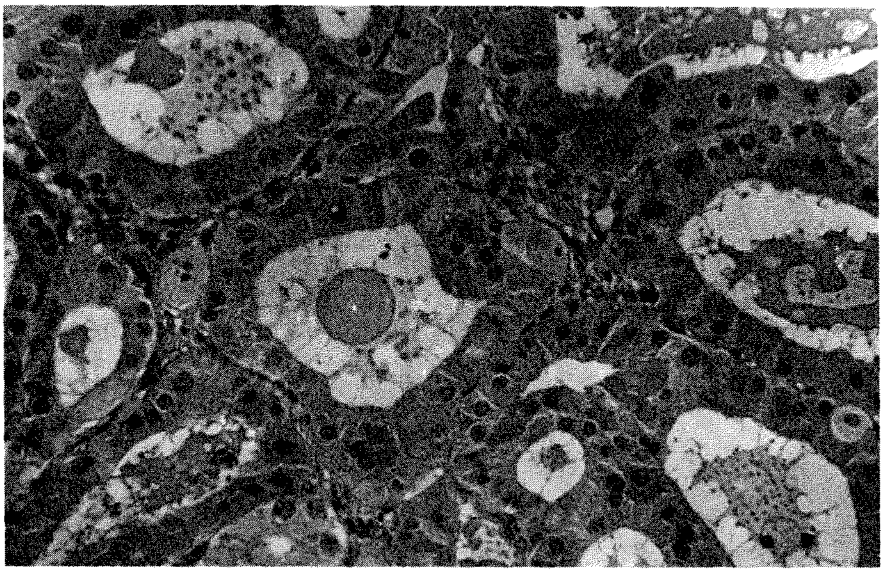

**Figure 3** Microphotography of the intraoperative biopsy of liver metastasis (immunohistochemistry with somatostatin antiserum).

### CONCLUSIONS

Somatostatinoma is seldom diagnosed preoperatively: physical examination, clinical symptoms and imaging techniques do not give specific results. Duodenal types are generally asymptomatic from the hormonal aspect and may not produce, as in the case we reported, high levels of circulating somatostatin.

The prognosis of resectable somatostatinoma is much better than that described in pancreatic and biliary adenocarcinomas, so the main goal is to achieve, mainly with immunohistochemistry, a correct diagnosis, in order to plan effective surgery. Cytotoxic therapy may be given in diffuse neoplasms with acceptable results.

### REFERENCES

1. Pearse, A. G. E. (1974) The APUD cell concept and its implications in pathology. *Path. Annu.* **9**, 27–41.
2. Ganda, O. P., Weir, G. C., Soeldner, J. S., Legg, M. A., Chick, W. L., Patel, Y. C., Ebeid, A. M., Gabbay, K. H. and Reichlin, S. (1977) Somatostatinoma: somatostatin- containing tumor of the endocrine pancreas. *N. Eng. J. Med.* **296**, 963–967.
3. Larsson, L. I., Hirsch, M. A., Holst, J. J., Ingemansson, S., Kuhl, C., Lindkaer Jenssen, S., Lundqvist, G., Rehfeld, J. F. Schartz, T. W. (1977) Pancreatic somatostatinoma. Clinical features and physiological implications. *Lancet* **1**, 666–668.
4. Kaneko, H., Yanaihara, N., Ito, S., Kusumoto, Y., Fujita, T., Ishikawa, S., Sumida, T. and Sekya, M. (1979) Somatostatinoma of the duodenum. *Cancer* **44**, 2273–2279.
5. Kaneko, H., Toshima, M., Kobayashi, H., Kitazawa, M., Ito, S., Iwanaga, T., Kusumoto, Y., Fujita, T. and Nitta, H. (1983) Duodenal somatostatinoma Immunohistopathology and review of the literature. *Acta Path. Jap.* **33**, 153–158.
6. Krejs, G. J., Orei, L., Conlon, J. M., Ravazzola, M., Davis, G. R., Raskin, P., Collins, S. M., McCarthy, D. M., Baetens, D., Rubenstein, A., Aldor, T. A. M. and Unger, R. H. (1979) Somatostatinoma syndrome. Biochemical, morphological and clinical features. *N. Eng. J. Med.* **301**, 285–292.
7. Harris, G. J., Tio, F. and Cruz, A. B. (1987) Somatostatinoma: a case report and a review of the literature. *J. Surg. Onco.* **36**, 8–16.
8. Jensen, S. L.,Christiansen, L. A., Oxholm, A. M. and Holst, J. J. (1984) Duodenal somatostatinoma. *Brit. J. Surg.* **71**, 159.
9. Somers, G., Pipeleers-Marichal, M., Gepts, W. and Pipeleers, D. (1983) A case of duodenal somatostatinoma: diagnostic usefulness of calcium-pentagastrin test. *Gastroentero.* **85**, 1192–1198.
10. Pipeleers, D., Couturier, E., Gepts, W., De Nutte, N. and De Vroede, M. (1979) Plasma pancreatic hormone levels in a case of somatostatinoma. Diagnostic and therapeutic implications. *J. Clin. Endocrin. Metabol.* **49**, 572–579.
11. Roberts, L. Jr., Dunnick, N. R., Foster, W. L. Jr. and Thompson, W. M. (1984) Somatostatinoma of the endocrine pancreas: CT findings. *J. Comp. Assist. Tomo.* **8**, 1015–1018.

